# Multi-omics landscape of Interferon-stimulated gene OASL reveals a potential biomarker in pan-cancer: from prognosis to tumor microenvironment

**DOI:** 10.3389/fimmu.2024.1402951

**Published:** 2024-09-02

**Authors:** Yi Liu, Runyu Yang, Mengyao Zhang, Bingyu Yang, Yue Du, Hui Feng, Wenjuan Wang, Busheng Xue, Fan Niu, Pengcheng He

**Affiliations:** Department of Hematology, The First Affiliated Hospital of Xi’an Jiaotong University, Xi’an, Shaanxi, China

**Keywords:** OASL, pan-cancer analysis, tumor immune microenvironment, T cell dysfunction, biomarker

## Abstract

**Background:**

OASL (Oligoadenylate Synthetase-Like), an interferon-induced protein in the OAS family, plays a significant role in anti-viral response. Studies have demonstrated its association with prognosis of certain tumors. However, the mechanism through which OASL affects tumors is unclear. A systemic pan-cancer study of OASL needs to be illustrated.

**Methods:**

Analysis of OASL expression across 33 tumors was conducted utilizing TCGA, GTEx and CPTAC databases. COX and Log-Rank regressions were employed to calculate the prognosis. We validated the impact of OASL on apoptosis, migration, and invasion in pancreatic cancer cell lines. Moreover, we employed seven algorithms in bulk data to investigate the association of OASL expression and immune cell infiltration within tumor immune microenvironment (TIME) and ultimately validated at single-cell transcriptome level.

**Results:**

We discovered elevated expression of OASL and its genetic heterogeneity in certain tumors, which link closely to prognosis. Validation experiments were conducted in PAAD and confirmed these findings. Additionally, OASL regulates immune checkpoint ligand such as programmed death ligand 1 (PD-L1), through IFN-γ/STAT1 and IL-6/JAK/STAT3 pathways in tumor cells. Meanwhile, OASL affects macrophages infiltration in TIME. By these mechanisms OASL could cause dysfunction of cytotoxic T lymphocytes (CTLs) in tumors.

**Discussion:**

Multi-omics analysis reveals OASL as a prognostic and immunological biomarker in pan-cancer.

## 1 Introduction

Cancer remains a serious threat to human health and life over past years (1). The emergence of immunotherapy represents a major shift from traditional cancer treatments, offering hope to patients with advanced cancer (2, 3). However, immunotherapy represented by immune checkpoint blockers (ICBs) cannot benefit some patients and cannot provide long-term effects (4–6). This is largely due to the immunosuppressive effects within the tumor immune microenvironment (TIME) (7, 8). TIME is a complex biological network comprising tumor cells, immune cells (such as T cells, B cells, macrophages and natural killer/NK cells), stromal cells, endothelial cells, and their secreted signaling molecules (including cytokines, chemokines and growth factors) (9). Numerous immune cells exhibit dual roles of promoting or inhibiting tumors (10). In this condition, cells and molecules in TIME dynamically evolved, leading to the accumulation of immunosuppressive cells like myeloid-derived suppressor cells (MDSCs), regulatory T cells (Tregs), tumor-associated macrophages (TAMs), and pro-inflammatory factors such as IL-6, IL-10, TGF-β (11, 12). Concurrently, the upregulation of immune checkpoints on T cells, such as programmed death 1(PD-1) and CTLA-4, enhances binding with programmed death ligand 1 (PD-L1), inducing T cell dysfunction or exhaustion, thereby effectively suppressing anti-tumor responses and facilitating immune escape (13–15). Although increasing evidence elucidates the significant role of TIME in inhibiting tumor immunity, our understanding of the mechanisms behind immune escape remains limited. Thus, further clarification of their interactions is urgently needed to inform new cancer immunotherapy approaches.

OASL (Oligoadenylate Synthetase-Like) is an interferon-induced protein belonging to the OAS family, which also includes OAS1, OAS2, and OAS3 (16). Unlike other OAS members, OASL lacks 2’, 5’-oligoadenylate synthetase activity and mediates antiviral responses through a non-classical pathway that does not rely on RNase L (17–20). Recent research has found the catalytic core of cyclic GMP-AMP synthase (cGAS) to be structurally homologous to the RNA-sensing enzyme, 2’-5’ oligo-adenylate synthase (OAS) (21). Structural and functional analyses have shown that OASL’s antiviral activity is enhanced by binding to dsRNA, thereby augmenting the signaling of the retinoic acid-inducible gene-I (RIG-I) RNA sensor (20, 22). Gene variations of OASL are associated with the occurrence and development of various diseases. Previous studies have linked its single nucleotide polymorphisms (SNPs) with responses to viral infections. *Su’s* research indicated that OASL SNPs (rs3213545, rs1169279, rs2859398) are involved in the host response to IFN treatment in hepatitis C virus (HCV) patients (23). *Lopez-Rodriguez et al.* found that OASL polymorphism (rs12819210) is an independent predictor of sustained virological response (SVR) in HCV (24). Genomic study from *Choi et al.* demonstrated that SNPs of OASL (rs1169279 and rs3213545) affect various cardiovascular-related diseases (20). Additionally, as one of interferon-stimulated gene (ISGs), OASL has been reported to play a crucial role in the development of many autoimmune diseases. OASL variants (such as R60W, T261S, A447V) significantly accumulate in patients with systemic lupus erythematosus (SLE), promoting the secretion of IFN-α (25). OASL may also serve as a predictive biomarker for assessing rheumatoid arthritis (RA) patients’ response to tocilizumab (26). At the protein level, overexpression of OASL upregulates TET1 through IRF1 signaling, inducing aberrant activation of CD4+ T cells in systemic sclerosis (27).

Even contributing significantly to anti-viral immunity, studies of OASL in tumors has become a focus of attention recently. *Zhao et al.* demonstrated that OASL is overexpressed in stomach adenocarcinoma (STAD) tissues and cell lines, which promoting proliferation, migration, invasion, and tumor formation through mTOR signaling pathway (28). Similarly, in basal-like breast cancer (BLBC) and pancreatic ductal adenocarcinoma (PDAC), analysis of bioinformatics mining has shown that highly expressed OASL is associated with poor prognosis (29–31). However, OASL’s overexpression indicates better overall survival for patients in bladder urothelial carcinoma (BLCA), and is associated with the infiltration levels of CD4+ T cells, CD8+ T cells, neutrophils, and dendritic cells (32). These results suggest that promotive or inhibitory effects of OASL depend on the cancer type, indicating a possible association with specific TIME signature. Despite increasing reports has been put on OASL’s role in cancer, there is currently no systematic study on OASL in pan-cancer, especially regarding aspects related to TIME.

**Figure 1:**
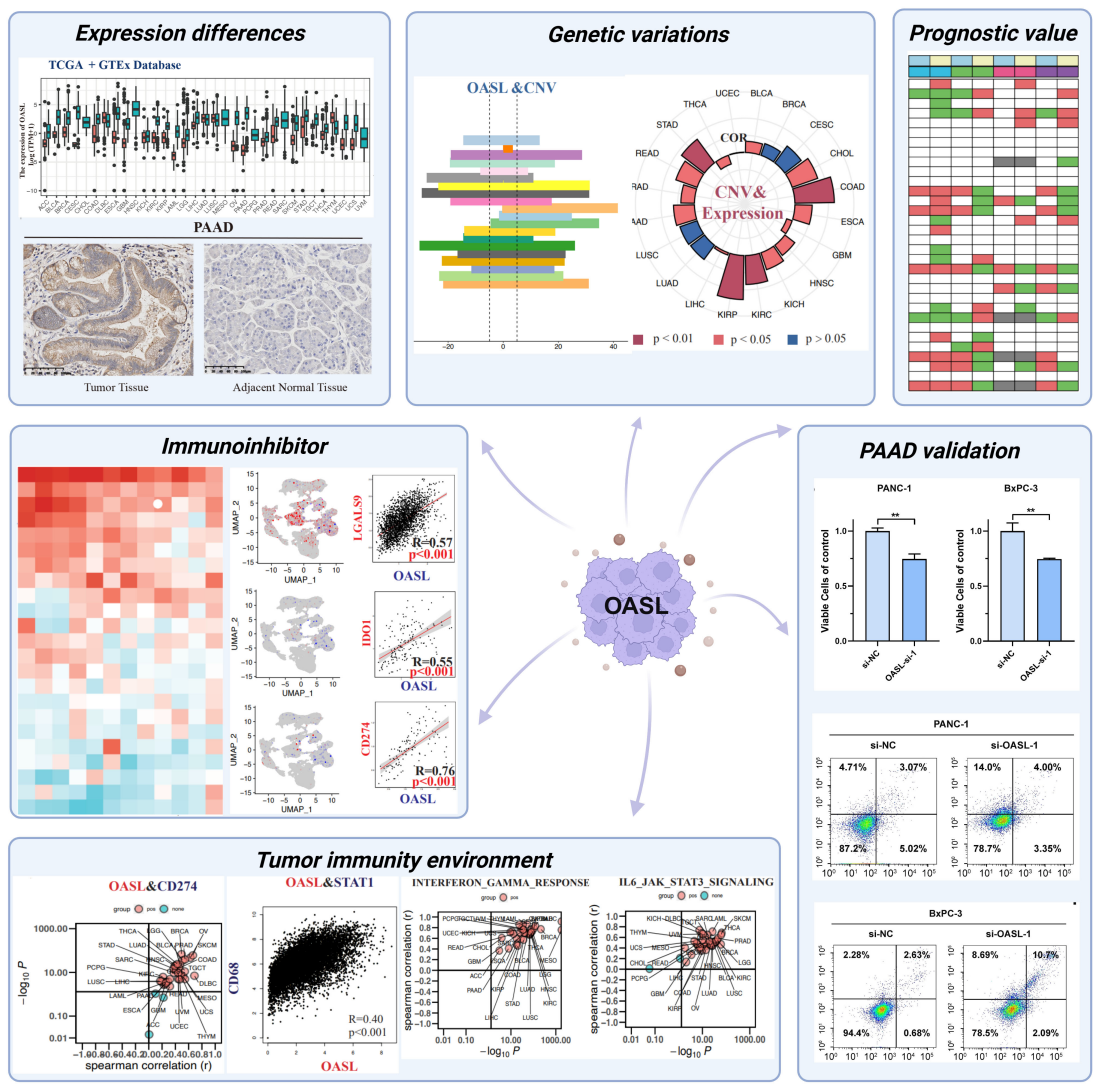
Schematic diagram illustrating pan-cancer analysis of the OASL gene in this study. The diagram was created with BioRender.com. (Mean ± SD, n = 3; ** indicates p < 0.01)

In our study, we systematically investigated the OASL gene in pan-cancer through the integration of multi-omics data and bioinformatics analysis. We found that OASL overexpressed significantly across most of tumors and has prognostic implications. To further validate these findings, *in vitro* experiments were conducted in two cell lines of pancreatic adenocarcinoma (PAAD) and with our cohort. Moreover, we examined the influence of OASL on genomic stability and genetic variability in various cancers. Importantly, our research revealed that overexpression of OASL could induce cytotoxic T lymphocyte (CTL) dysfunction by upregulating immune checkpoint ligands, such as PD-L1, and inducing macrophage immune infiltration in the TIME (**Figure 1**). In summary, OASL remains a complex and active role in cancer research. Researchers are striving to explore its specific mechanisms of immune response within TIME, aiming to inform immunotherapy approaches for cancer.

## 2 Materials and methods

### 2.1 OASL expression in human cancers

To explore the role of OASL in human cancers, we analyzed gene transcriptome data in TPM- format for 33 types of human tumors. We accessed the “Gene_DE” module within the Tumor Immune Estimation Resource TIMER ( *http://timer.cistrome.org/* ) and performed a comparative study on the expression of the OASL at mRNA level across various tumor types. Combined with Cancer Cell Line Encyclopedia (CCLE) and Genotype-Tissue Expression (GTEx) project, the pan-cancer sample data were acquired from TCGA database, were retrieved *via* the UCSC Xena browser ( *https://xenabrowser.net/datapages/* ). Using R software (*version* 4.1.2), we employed the Wilcoxon rank sum test to analyze the disparities in OASL expression between tumor tissues and normal tissues. The variations in expression levels were then visualized through ggplot2 (*version* 3.4.2). Additionally, we utilized the Clinical Proteomic Tumor Analysis Consortium (CPTAC, *https://pdc.cancer.gov/pdc/* ) to compare the protein expression of OASL across various tumor types at the proteomic level. We also conducted a comparative study on the expression of OASL across various tumor stages using the Gene Expression Profiling Interactive Analysis (GEPIA, *http://gepia2.cancer-pku.cn/* ). The Human Protein Atlas (HPA; *www.proteinatlas.org* ), a comprehensive database on human protein expression and distribution, along with tissue microarrays, indicated differential OASL expression in tumor versus normal tissues through immunohistochemistry. Lastly, bubble maps detailing OASL-related diseases were extracted from the Open Target Platform ( *https://platform.opentargets.org/* ) to explore potential associations between OASL expression and disease.

### 2.2 Survival analysis

The expression profile of TCGA pan-cancer transcriptome data comes from The Pan-Cancer Atlas ( *https://gdc.cancer.gov/about-data/publications/pancanatlas* ), and the patient survival data comes from UCSC Xena browser, using the R package survival (*version* 3.5-5) and survminer (*version* 0.4.9) to perform a univariate COX model to calculate the risk based on the continuous value of OASL expression. At the same time, based on the OASL expression, calculate the optimal cutoff value and perform Kaplan-Meier risk calculation. All graph visualizations are based on ggplot2 (*version* 3.4.2). Disease free interval (DFI), disease specific survival (DSS), overall survival (OS), and progression free interval (PFI). The hazard ratio (HR), representing the relative risk between high and low OASL expression groups, was calculated to determine OASL’s influence on survival outcomes.

### 2.3 Genetic variations analysis of OASL

For the analysis of gene mutations, the Simple Nucleotide Variation dataset, processed by MuTect2 software, was procured from the Genomic Data Commons (GDC, *https://portal.gdc.cancer.gov/* ). In order to analyze the copy number variant (CNV) level of the OASL gene, we paid special attention to the CNVs in the region where the gene is located. We integrated the mutation data and acquired structural domain information for the proteins utilizing the R package maftools *(version* 2.2.10). Through integrating genomic heterogeneity analysis with previous gene expression data, we calculated the correlation between OASL expression and homologous recombination deficiency (HRD), loss of heterozygosity (LOH), mutant-allele tumor heterogeneity (MATH), microsatellite instability (MSI), ploidy, purity, tumor mutation burden (TMB), *etc.*.

### 2.4 Investigating tumor immune microenvironment of OASL

We employed the ESTIMATE R package (*version* 1.0.13) to calculate stromal, immune, and purity (ESTIMATE) scores for patients across different tumors. The deconvo_tme function of the IOBR package (*version* 0.99.9), incorporating the xCell algorithm, was used to reassess major immune cell infiltration scores *via* gene expression analysis. For a detailed assessment of M2 macrophage infiltration, we applied both CIBERSORT and QUANTISEQ algorithms. The correlation between OASL expression and immune cell infiltration was determined through Pearson correlation analysis, calculating the correlation coefficient. The prognostic relevance of OASL expression and cytotoxic T lymphocyte (CTL) infiltration was examined using the Tumor Immune Dysfunction and Exclusion (TIDE, *http://tide.dfci.harvard.edu* ) database. Furthermore, the predictive value of OASL for immunotherapy efficacy was analyzed in comparison with other indicators *via* the TIDE database.

### 2.5 OASL expression at the single-cell resolution

Further validation of the relationship between OASL and immune biomarkers was accomplished *via* single-cell transcriptome data from Tumor Immune Single-cell Hub (TISH, *http://tisch.comp-genomics.org/home/* ) database. R package Seurat (*version* 4.3.0) was employed for data processing. To examine gene expression patterns, the “FeaturePlot” function illustrated the co-expression of two genes, and “getScatterplot” was employed to analyze and visualize the correlation between the expressions of two distinct genes at the single-cell level.

### 2.6 Cell culture

PANC-1 and BxPC-3 cell lines were purchased from ATCC^®^. Cells were cultured in DMEM medium (Gibco^®^) supplemented with 10% of fetal bovine serum (FBS, Gibco^®^). Adherent cultivation was maintained with a passage ratio of 1:3 ~ 1:4, under condition of 37°C and 5% CO_2_.

### 2.7 Transfection of small interfering RNA

PANC-1 and BxPC-3 cells were seeded in a 6-well plate, and transfection was initiated when cell confluence reached approximately 60 ~ 70%. The cell culture medium was replaced with 2% FBS. The designed sequences for siRNA-NC, OASL-1, OASL-2 and OASL-3 were displayed in **Supplementary Table S1**. Subsequently, siRNA (10 nM, 4 μl) and Lipofectamine™ RNAiMAX (Invitrogen^®^) transfection reagent (12 μl) were added to 100 μl of Opti-MEM medium (Gibco^®^) for the transfection system A and B, respectively. The mixture was allowed to stand at room temperature for 5 minutes. Afterward, the two liquid components (A, B) were gently mixed, avoiding vigorous shaking, and left to stand for additional 15 minutes. The transfection system was evenly applied to the cells in the 6-well plate, and the medium was replaced with complete culture medium (10% FBS) after 6 hours incubation. Cells were harvested 24 hours post-transfection.

### 2.8 Estimation of siRNA knockdown efficacy by quantitative PCR

The mRNA was extracted from the cells post-transfection, and the resulting products were converted into cDNA using the Takara Reversal Kit^®^. For each sample, 50 ng of cDNA was utilized for qPCR assay to verify the efficacy of knockdown by si-RNA. The specific primers sequences were detailed in the **Supplementary Table S2**. All the aforementioned reagents were procured from Tsingke Biotechnology Co., Ltd.

### 2.9 Invasion tests

Prepare the matrix adhesive Matrigel (Thermo Fisher Scientific^®^), transwell chamber (Corning^®^), and a 24-well plate. Pre-cool the Matrigel with serum-free medium, mix it thoroughly, and evenly spread it on the bottom of the chamber. Incubate the Matrigel in an incubator (37°C, 5% CO_2_) for 3 hours to solidify. Before preparing the cell suspension, starve the cells of serum for 12~24 hours to further eliminate serum influence. Digest cells with a confluence of 70 ~ 80%, centrifuge them, and re-suspend the cells in serum-free medium, meanwhile adjusting the cell density to 2.5×10^5^/ml after counting. Place the chamber in the 24-well plate after adding 500 μl of complete medium to the lower plate. Inoculate the upper chamber with 200 μl of cell suspension for 24 hours, followed by fix the cells with paraformaldehyde in the 24-well plate. Discard the fixing solution in the 24-well plate, wash with PBS, stain with 0.1% crystal violet for 5 ~ 10 minutes. Wash with PBS again to remove unbound crystal violet, and then observe and count the cells in three fields by a microscope after air-drying.

### 2.10 Cell viability assays

Post-transfection cells were seeded into a 96-well plate with three independent samples. After co-culturing for 24 hours, 10 µl CCK-8 (DoJindo^®^ Laboratories) was added into each well followed by an incubation of 2 ~ 3 hours. The absorbance of each well at 450 nm and 690 nm wavelength was measured with a microplate reader. Viable cells were calculated following the instructor’s protocol, viable cells = (test group OD_450_-OD_690_/untreated group OD_450_-OD_690_) ×100%.

### 2.11 Apoptosis detection

Cells post-transfection were collected and centrifuged prior, followed by washing with PBS for twice. Apoptosis Reagent Kit was purchased from Biolegend^®^. Annexin V (5 μl) and PI antibodies (10 μl) were added into 200 μl of binding buffer for the detection solution. After co-incubitaed for 20 minutes at room temperature, the experiments were conducted *via* a BD FACS Canto II^®^ flow cytometer. Annexin V+/PI- or Annexin V-/PI+ cells were defined as apoptotic cells. Data was processed by FlowJo and GraphPad software.

### 2.12 Immunohistochemistry staining of patient samples

Nine pancreatic cancer patient samples were obtained from the pathology department of the First Affiliated Hospital of Xi’an Jiaotong University. All samples were confirmed as pancreatic ductal carcinoma through tissue biopsy. The primary antibodies used included a rabbit monoclonal antibody against OASL (Abcam^®^, ab229136). All stained sections were scanned panoramically and scored using Image J profile.

### 2.13 Statistical analysis

As for cell viability assays, the values of each group were normalized to the control group. In qPCR quantification, “2^–ΔΔCt^” is a method used to calculate the relative expression level of a specific gene in a sample, while normalization was performed to the corresponding control group. Regarding as apoptotic detection, the ratio of apoptotic cells in each treatment group was normalized to the control group. IHC scoring analysis was determined according to the following criteria, the positive area (A) was assigned a numerical score ranging from 1 to 4: 1 for positive area < 10%, 2 for positive area 20 ~ 50%, 3 for positive area 50 ~ 80%, and 4 for positive area > 80%; in terms of intensity (I), a numerical score ranging from 1 to 3 was employed: 1 for positive intensity (+), 2 for positive intensity (++), and 3 for positive intensity (+++); the total score, A × I, was calculated accordingly, and statistical analysis was performed using a two-tailed unpaired Student’s *t*-test. These quantification results were presented as the Mean ± SD. Statistical analysis involved a two-tailed unpaired Student’s *t*-test for two groups, and one-way ANOVA test followed by Tukey’s *post-hoc* comparison for multiple subgroups. A value of *p* < 0.05 was considered as significantly difference, * indicates *p* < 0.05, ** indicates *p* < 0.01, *** indicates *p* < 0.001, **** indicates *p* < 0.0001, ns stands for not statistically significant. GraphPad 8.0 was used for statistical analysis.

### 2.14 Ethics statement

Ethical approval for studies involving human participants was obtained from The Ethics Committee of the First Affiliated Hospital of Xi’an Jiaotong University (XITU1AF2022LSK-339).

## 3 Results

### 3.1 The expression level of OASL in various tumors

**Figure 2:**
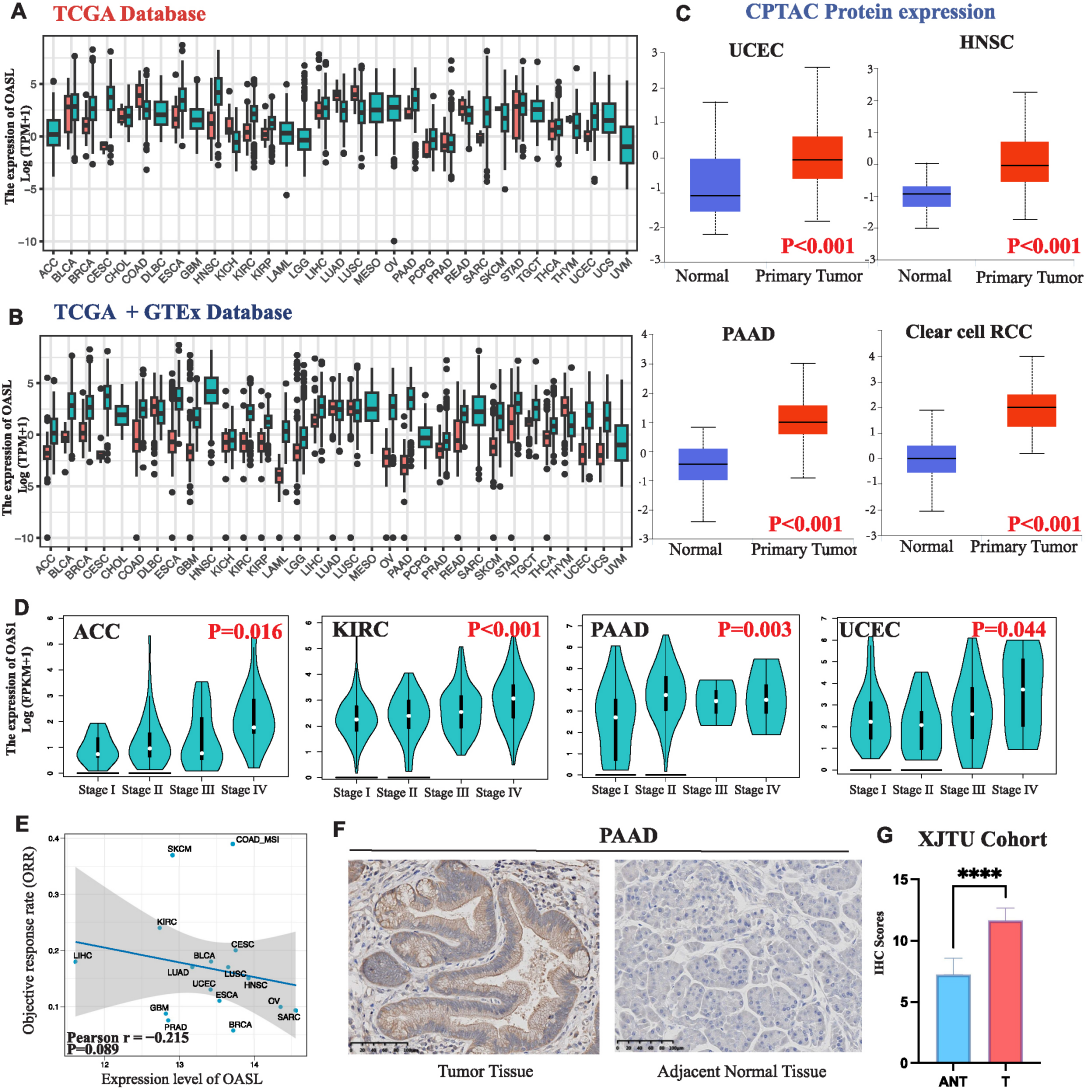
OASL expression in various cancers. **(A)** OASL was highly expressed in a variety of tumors compared to normal tissues in TCGA database. **(B)** OASL expression in TCGA combined with GTEx databases. **(C)** OASL protein was highly expressed in UCEC, HNSC, PAAD, and Clear cell RCC in the proteomic database CPTAC. **(D)** OASL expression in different pathological grades (Stage I, II, III and IV) of ACC, KIRC, PAAD, UCEC in GEPIA2 database. **(E)** The expression of OASL was negatively correlated with the ORR in cancers (Pearson r = -0.215, *p* = 0.089). **(F)** IHC staining validated in PAAD showed that OASL expression was significantly elevated comparing to adjacent normal tissue. **(G)** The statistics of IHC score in XJTU cohort of PAAD clinical samples. These quantification results were presented as the (Mean ± SD, n = 9; **** indicates *p* < 0.0001).

To gain a deeper insight into OASL’s function in various cancers, we examined its expression patterns using The Cancer Genome Atlas (TCGA) database, which encompasses detailed descriptions on 33 different cancer types (**Supplementary Table S3**). Firstly, we compared the mRNA expression of OASL in normal and tumor tissues using the TCGA database, revealing high expression in ESCA, HNSC, KIRC, KIRP, PAAD, PCPG, and UCEC (**Figure 2A**). Due to the absence of some normal tissue data in the TCGA database as a control, we integrated data from the GTEx database. The results still showed high expression in ACC, BLCA, BRCA, CESC, COAD, GBM, LAML, LGG, OV, READ, SKCM, STAD, TGCT, THCA, and UCS (**Figure 2B**). The CPTAC proteomic data demonstrated a high expression in UCEC (*p <* 0.001), HNSC (*p <* 0.001), PAAD (*p <* 0.001), and Clear cell RCC tumors (*p <* 0.001) at the proteomic level (**Figure 2C**). Moreover, OASL expression is positively associated to the pathological grades across tumors, especially in ACC (*p =* 0.016), KIRC (*p <* 0.001), PAAD (*p =* 0.003), and UCEC (*p* = 0.044), as shown in **Figure 2D**. Furthermore, Pearson analysis revealed correlations between OASL expression and the objective response rate (ORR) in some tumors (r = -0.215, *p* = 0.089) (**Figure 2E**). This finding suggests a potential link between higher OASL expression and increased treatment resistance, as well as the prognosis of these tumors. Additionally, immunohistochemistry (IHC) verification was performed in pancreatic adenocarcinoma (PAAD), which consisting of 9 samples, and the pathological samples analyzed were exclusively sourced from the newly diagnosed PAAD patients at the First Affiliated Hospital of Xi’an Jiaotong University (XJTU cohort) (**Figure 2F**). Utilizing a double-blind approach, we evaluated the stained pathological sections, as the statistical outcomes presented in **Figure 2G**, which revealing OASL expression was significantly elevated in PAAD tissue compared to adjacent non-cancerous tissue (*p* < 0.0001).

### 3.2 Evaluation of the correlation between OASL expression and prognosis

**Figure 3:**
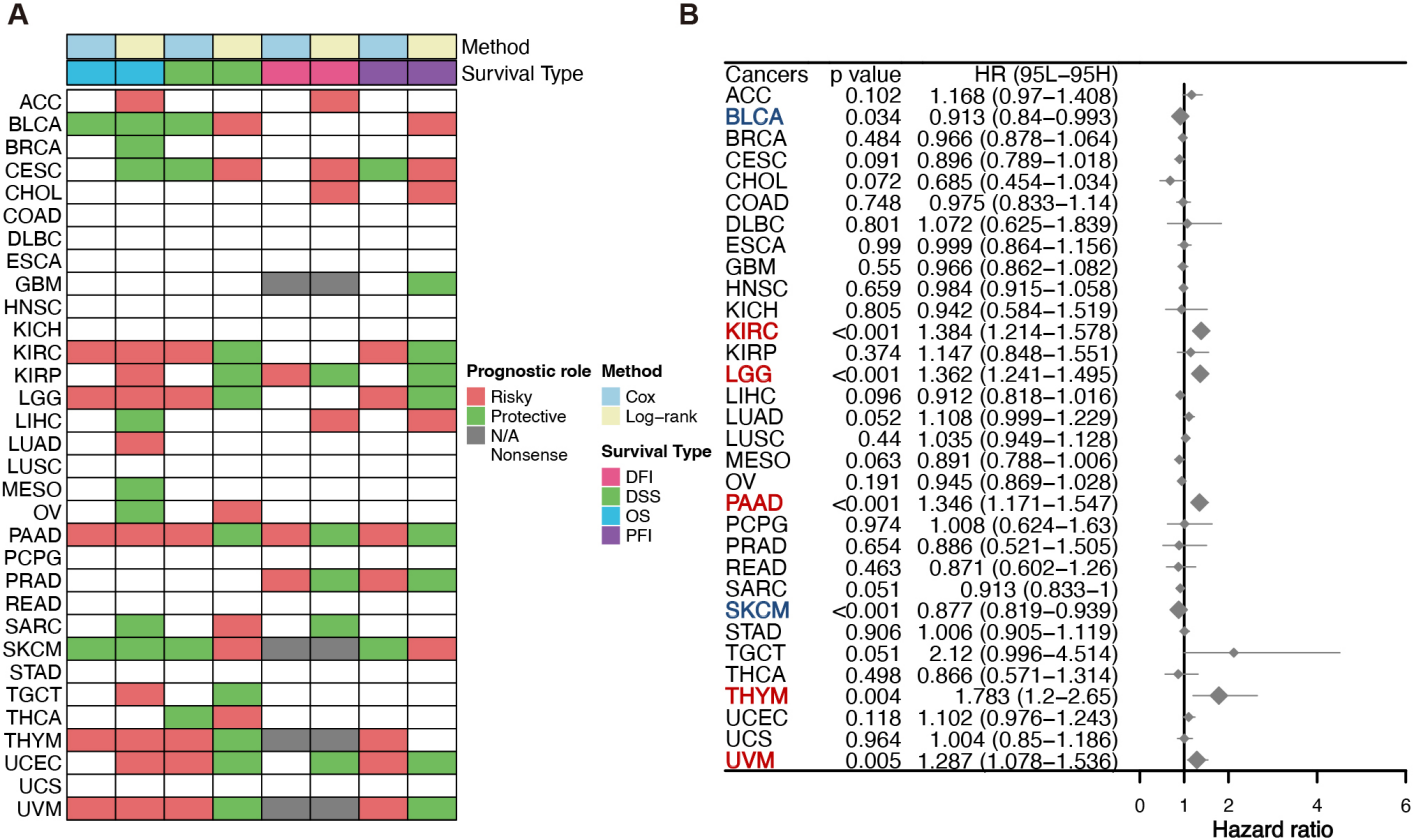
Survival evaluation of OASL expression. **(A)** Various prognostic indicators such as DFI, DSS, OS and PFI in different tumors were evaluated by two distinct statistical methods (COX and Log-rank) in TCGA database. **(B)** COX regression was employed to evaluate OS in diverse tumor types, and the results indicated statistically significant prognostic implications in KIRC (*p* < 0.001, HR = 1.384), LGG (*p* < 0.001, HR = 1.362), PAAD (*p* < 0.001, HR = 1.346), THYM (*p* = 0.004, HR = 1.783), UVM (*p* = 0.005, HR = 1.287), BLCA (*p* = 0.034, HR = 0.913) and SKCM (*p* < 0.001, HR = 0.877).

To evaluate patient survival, we applied COX and Log-rank regression models for an initial examination of the association between OASL expression and prognostic factors such as DFI, DSS, OS, and PFI across 33 tumor types. As shown in **Figure 3A**, OASL plays varying roles in survival outcomes. To delve deeper, we specifically focused on OS, the most prevalent prognostic indicator in clinical settings, employing the COX-regression method with HR for detailed survival analysis. Current analysis reveals that increased OASL expression is associated with worse prognosis in KIRC (*p* < 0.001, HR = 1.384), LGG (*p* < 0.001, HR = 1.362), PAAD (*p* < 0.001, HR = 1.346), THYM (*p* = 0.004, HR = 1.783), and UVM (*p* = 0.005, HR = 1.287). Conversely, OASL expression appears to be a protective factor in BLCA (*p* = 0.034, HR = 0.913) and SKCM (*p* < 0.001, HR = 0.877) (**Figure 3B**). The contrasting impact of OASL observed in certain tumor types is believed to be associated with their unique and specific molecular mechanisms. Therefore, further experimental validation is essential to confirm this observation.

### 3.3 OASL promotes cell proliferation and invasion in PAAD

**Figure 4:**
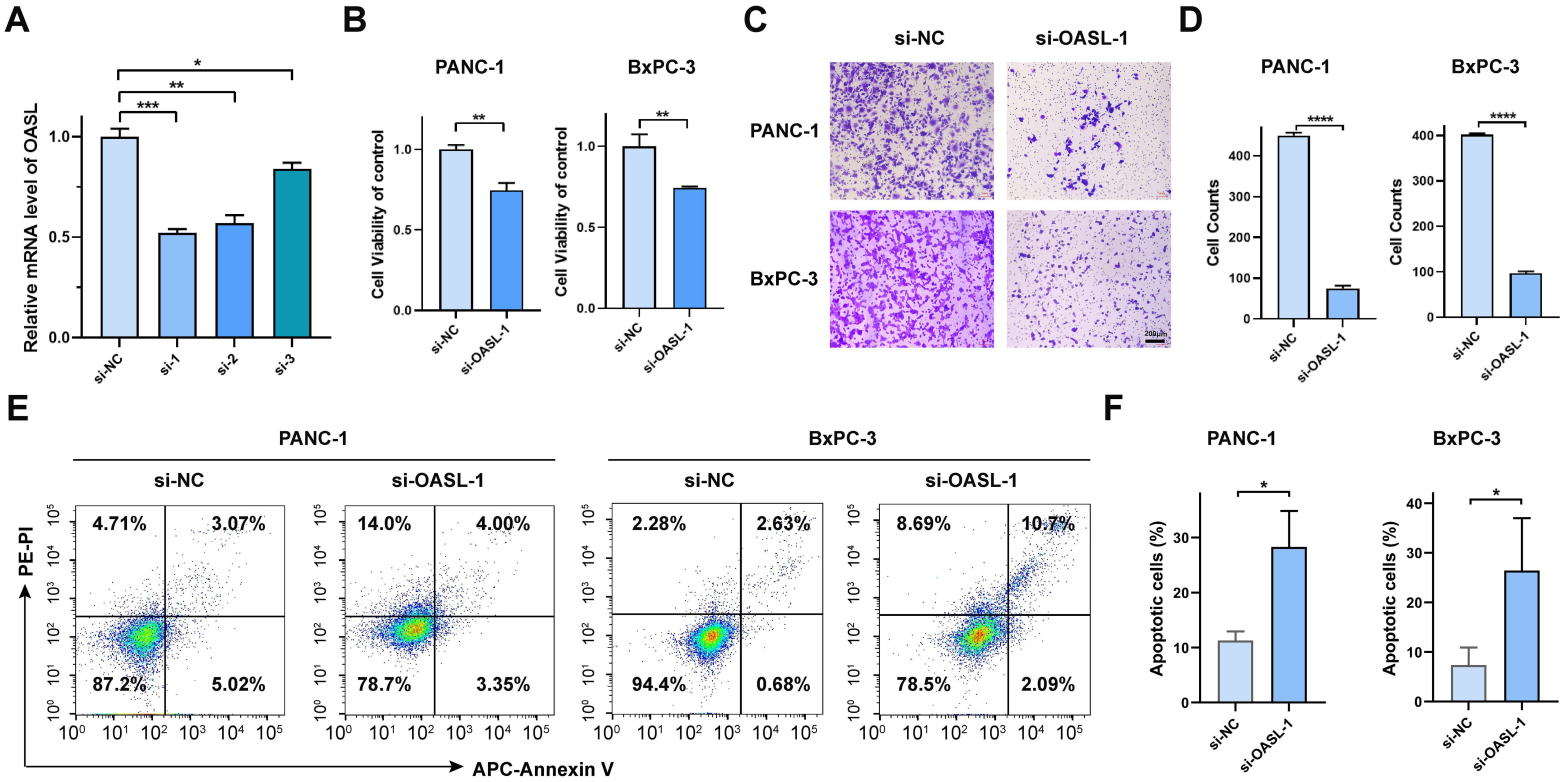
*In vitro* validation of OASL gene in pancreatic cancer cell lines. **(A)** Validation the efficiency of siRNA-OASL knockdown in mRNA level. **(B)** Cell viability analysis of PANC-1 and BxPC-3 following si-OASL-1 mediated knockdown. **(C, D)** The detection and statistics of invasion of PANC-1 and BxPC-3 after si-OASL-1 knockdown. **(E)** The apoptosis assays in OASL knockdown cell lines were tested by flow cytometer. **(F)** Statistical analysis of apoptotic cells in OASL knockdown PANC-1 and BxPC-3 cell lines (Mean ± SD, n = 3; * indicates *p* < 0.05, ** indicates *p* < 0.01, *** indicates *p* < 0.001, **** indicates *p* < 0.0001, ns stands for not statistically significant).

In order to ascertain the role of the OASL gene in tumors, with a particular emphasis on PAAD, our study utilized siRNA to perform knockdown experiments in the PANC-1 cell line. As illustrated in **Figure 4A**, the knockdown efficiency of si-OASL-1 compared to si-NC at the mRNA level was 52% ± 2% (*p* < 0.001), making it the chosen candidate for further experimental investigations. OASL gene knockdown led to a marked decrease of cell viability, as depicted in **Figure 4B**, 74.64% (*p* < 0.001) and 74.56% (*p* < 0.01) of the negative control in PANC-1 cells and in BxPC-3 cells, respectively. Transwell assays conducted on PANC-1 and BxPC-3 cell lines post-OASL knockdown revealed evidently decreased invasion capabilities. In PANC-1, invasion numbers fell from 450 ± 6.51 (si-NC) to 75 ± 6.66 (si-OASL-1) (*p* < 0.0001), and similarly in BxPC-3, from 402 ± 3.06 (si-NC) to 97 ± 4.13 (si-OASL-1) (*p* < 0.0001). These results highlight the crucial impact of OASL knockdown on diminishing the invasive potential of these pancreatic cancer cells (**Figures 4C, D**).

Additionally, apoptosis analysis was conducted *via* flow cytometry. The results indicated that knockdown of the OASL gene induces cell apoptosis, as illustrated in **Figures 4E, F**. Collectively, our *in vitro* experiments have demonstrated that OASL enhances the proliferative and invasive capabilities of PAAD cells. However, the precise mechanism by which OASL promotes PAAD cell growth remains unclear.

### 3.4 Genetic alteration analysis of OASL in distinct tumors

**Figure 5:**
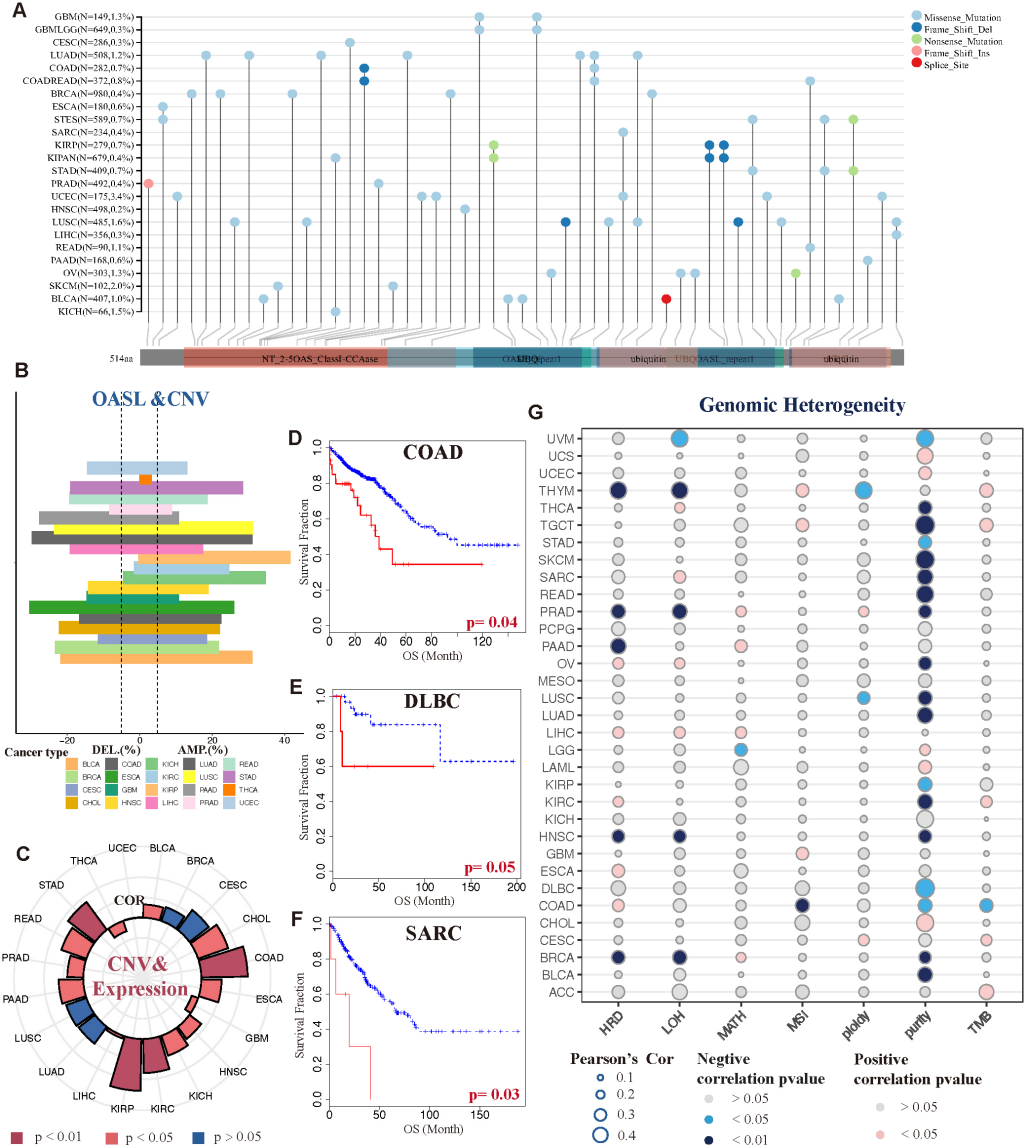
Genetic variations and heterogeneity of OASL. **(A)** The mutational status of the OASL gene across diverse tumor categories. **(B)** CNV gains were exhibited in various tumor types, such as KICH, KIRP, BLCA, LUSC and COAD. **(C)** The correlation between gains of CNV and expression of OASL. **(D-F)** The association between CNV level and prognostic survival (OS) in COAD (*p* = 0.04), DLBC (*p* = 0.05) and SARC (*p* = 0.03). **(G)** The correlation of genomic heterogeneity (HRD, LOH, MATH, MSI, ploidy, purity, TMB) in different tumors.

In terms of OASL genetic alterations in tumors, missense mutations are the predominant type, succeeded by frame-shift and nonsense mutations, whereas frame shift and splice alterations are less frequent. UCEC displayed the highest rate of gene mutations, reaching 3.4%. These mutations commonly take the form of missense mutations, which occur across various regions of the OASL gene. Additionally, this is followed by SKCM with a 2.0% rate of missense mutations, LUSC with 1.6% encompassing both missense mutations and frame shifts, KICH with 1.5% missense mutations, among others, as depicted in **Figure 5A**.

Moreover, we observed that copy number variation (CNV) in the form of amplifications or deletions exhibited variability across different tumor types, including BLCA, UCEC, KICH and COAD, as detailed in **Figure 5B**. Notably, BLCA, UCEC, and KICH primarily exhibited CNV amplifications, whereas COAD predominantly showed CNV deletions. This highlights the heterogeneity in OASL at the CNV level among these cancers. Furthermore, the extent of CNV variability was found to be closely linked to the expression levels of OASL, as shown in **Figure 5C**. Apart from a negative correlation and notable difference observed in THCA and GBM (*p* < 0.05), a positive correlation with high OASL expression was prevalent in most tumors, particularly in KIRP, COAD, STAD, and KIRC (*p* < 0.01). Most importantly, the variation in CNV level exhibited a significant association with OS, as demonstrated in cases of COAD (*p* = 0.04), DLBC (*p* = 0.05), and SARC (*p* = 0.03). In these particular tumor types, greater variability of CNV is linked to a more adverse prognosis (**Figures 5D-F**).

As illustrated in **Figure 5G**, the correlation of genomic heterogeneity across various tumors, encompassing factors such as HRD, LOH, MATH, MSI, ploidy, purity, TMB. Interestingly, we noted a pronounced and negative correlation between tumor purity and multiple cancer types (*p* < 0.05). The implication is that the lower the tumor purity, the more likely the tumor is to progress. In addition to tumor cells, purity reflects the presence of non-tumor cells within tumor tissues, such as immune cells and stromal cells, which collectively influence tumors’ development and progression. This concept aligns closely with that of the tumor microenvironment (TME). To a certain degree, this suggests that OASL’s impact on the onset and progression of various tumors could be linked to its role within the TME.

### 3.5 Correlations between OASL expression and TME in PAAD

**Figure 6:**
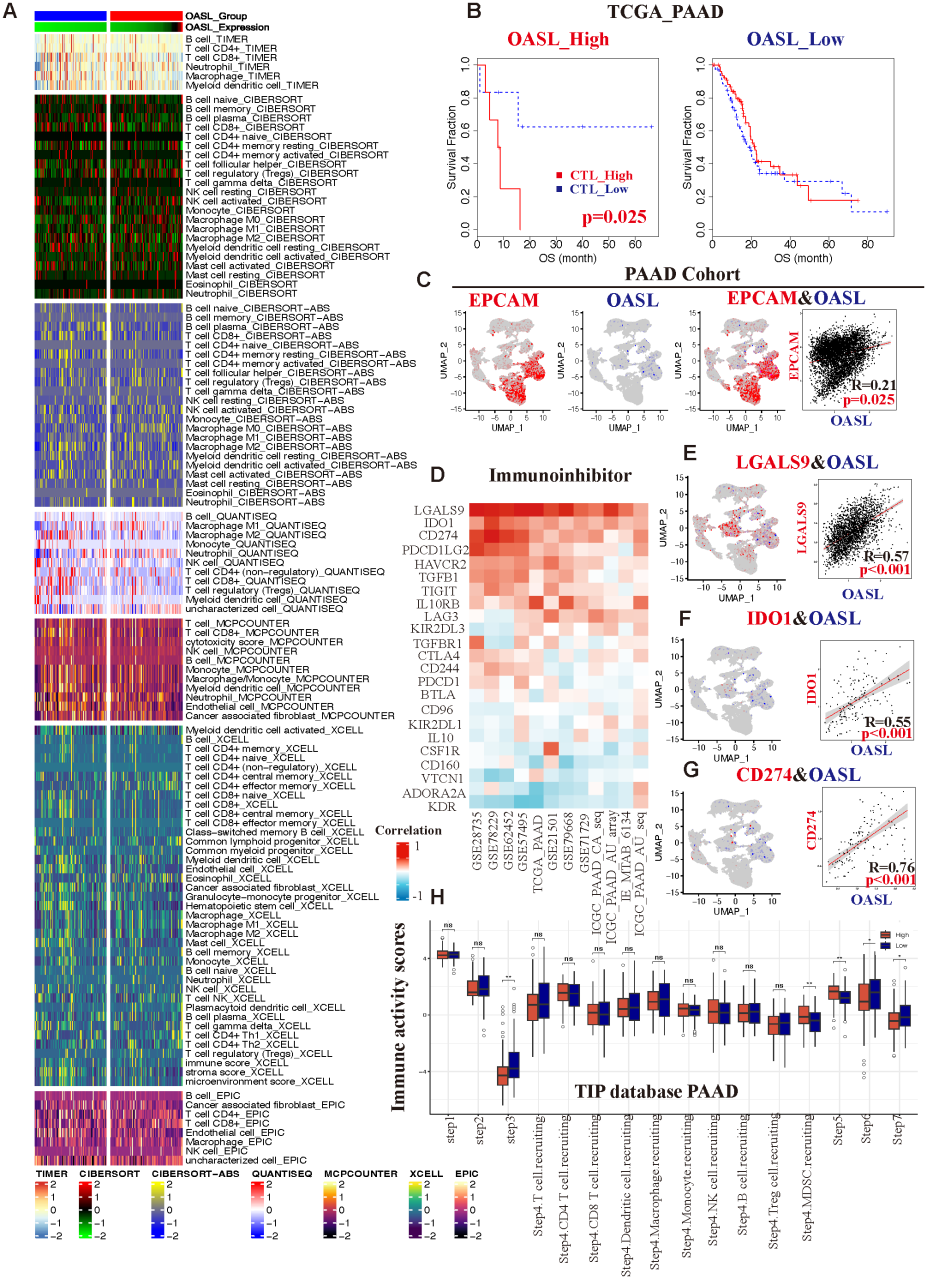
The correlation between OASL expression and TIME in PAAD. **(A)** Variety of algorithms targeted the relationship between different immune cells and OASL expression levels, and the results showed that CD8+ T cells played a crucial role in it. **(B)** In different groups of OASL expression, the amounts of infiltrated CTLs were associated with various prognosis. **(C)** An analysis of the correlation between EPCAM and OASL from a PAAD single cell database (R =0.21, *p* = 0.025). **(D)** The correlation of remarkable immune-inhibitors in distinct PAAD databases. The relationship from co-localization analysis between **(E)** OASL and LGALS9 (R =0.57, *p* < 0.001), **(F)** OASL and IDO1 (R =0.55, *p* < 0.001), **(G)** OASL and CD274/PD-L1 (R =0.76, *p* < 0.001). **(H)** Analysis of immune activity scores compared OASL_high to OASL_low expression group from TIP database in PAAD (* indicates *p* < 0.05, ** indicates *p* < 0.01, ns stands for not statistically significant).

Our previous findings confirmed the overexpression of OASL and its prognostic significance in PAAD, followed by an in-depth study of the role of OASL in the occurrence and progression of PAAD, with a focus on TME. A series of algorithms, including TIMER 2.0, CIBERSORT, CIBERSORT-ABS, QUANTISEQ, MCPCOUNTER, XCELL, and EPIC were employed to explore, as shown in **Figure 6A**. Across all datasets, there was a consistent observation of increased infiltration of CD8+ T cells (cytotoxic T lymphocytes, CTLs) and macrophages in the TME, particularly in the high OASL expression group. Building upon these results, we categorized CTL infiltration levels into two groups (CTL_high *vs.* CTL_low) within the TCGA database to conduct a prognostic analysis. Our findings revealed that in the high OASL expression group, the level of CTL infiltration markedly influenced OS (*p* = 0.025), meanwhile higher levels of CTL imply a poorer prognosis. Conversely, in the low OASL expression group, the impact of CTL infiltration on OS was not significant (**Figure 6B**). This differential effect underscores the importance of CTL infiltration levels in the prognosis of patients with varying OASL expression.

Furthermore, we verified the association between OASL and EPCAM in PAAD using a single-cell database (**Supplementary Table S4**). The epithelial cell adhesion molecule (EPCAM), also known as CD326, is widely expressed in various epithelial tissues and acts as a cell surface marker for numerous stem and progenitor cells. It is also recognized as an indicator of epithelial cell malignancy. Our analysis revealed a co-localization of expression between OASL and EPCAM, with the significant correlation (R = 0.21, *p* = 0.025), as illustrated in **Figure 6C**. Consequently, conclude that OASL is closely linked to the degree of tumor malignancy.

Next, we integrated of the GEO, TCGA, and ICGC databases, focusing on the immune-inhibitors study in PAAD. There were several key immune checkpoints such as LGALS9, IDO1, CD274 (PD-L1), PDCD1LG2, and HAVCR2, *etc.* (**Figure 6D**), and they were indicated a strong positive correlation with OASL expression in PAAD. To further demonstrate it, we employed single-cell database for verification revealing pronounced correlations specifically between OASL and LGALS9 (R =0.57, *p* < 0.001), IDO1 (R =0.55, *p* < 0.001), and PD-L1 (R =0.76, *p* < 0.001), as shown in **Figures 6E-G**.

As a novel tool for tumor immunity cycle analysis, this study utilized Tracking Tumor Immunophenotype (TIP) to assess the anti-tumor immune status at seven stages. This analysis indicates the proportion of immune cell infiltration and assigns an immune activity score during the anti-tumor process (33). In PAAD, OASL expression exhibited statistically significant differences at step 3 (priming and activation), part of step 4 (trafficking of immune cells to tumors - MDSC recruiting), step 5 (infiltration of immune cells into tumors), step 6 (recognition of cancer cells by T cells) and step 7 (killing of cancer cells). It well known that PD-L1 is indicative of an immunosuppressive state, and considering the established positive correlation between OASL and PD-L1 expression from our previous study. In our study, the group with low OASL expression displayed higher immune activity scores, showing a significant contrast to the high OASL expression group (**Figure 6H**). This suggests that elevated OASL expression may contribute to an immunosuppressive state especially in later stages of anti-tumor immunity process, such as during immune cell infiltration, T cell recognition, and cell killing.

### 3.6 Correlations between OASL expression and TME in pan-cancer

**Figure 7:**
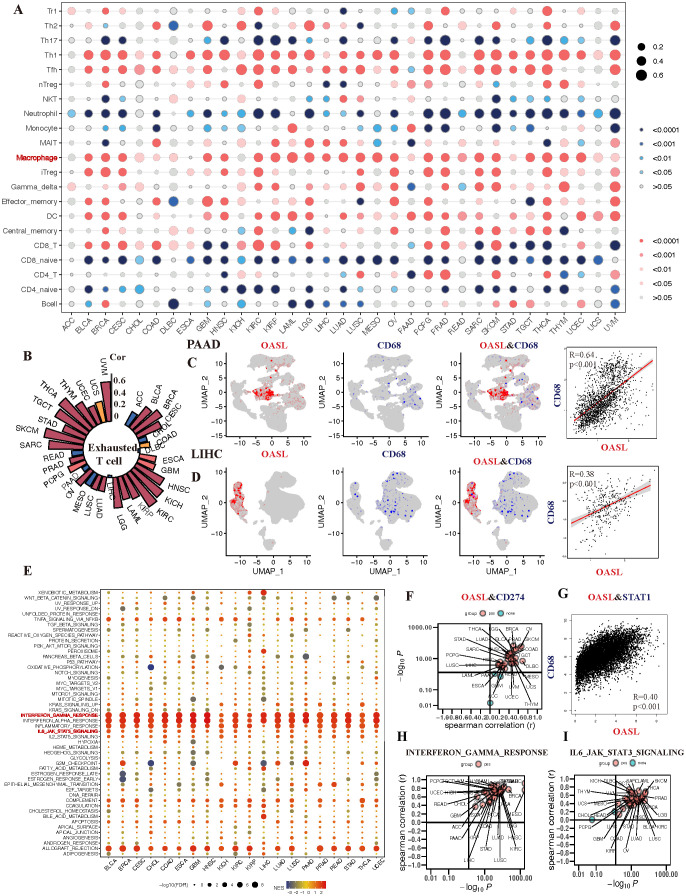
The association of OASL expression with TIME in various tumors. **(A)** The infiltration of macrophage is positively correlated with the development of almost tumors. **(B)** Analysis of a single cell sequencing database reveals that OASL expression is positively associated with exhausted T cell. **(C, D)** The expression of CD68 exhibits a certain co-localization and strong positive correlation with the expression of OASL in PAAD (R = 0.64, *p* < 0.001) and LIHC (R = 0.38, *p* < 0.001). **(E)** The correlation between the expression of OASL and the occurrence of immune-inflammatory mediators or signal pathways, including **(F)** CD274/PD-L1, **(G)** OASL and STAT1 (R = 0.40, *p* < 0.01), **(H)** OASL and IFN-γ response, **(I)** OASL and IL6_JAK_STAT3 signaling.

In our final analysis, we delved into the relationship between OASL expression and the TME across various cancers. Utilizing the XCELL algorithm, we further revealed that OASL expression positively correlates with macrophages, Th1 cells, and T follicular helper (Tfh) cells, while exhibiting a negative correlation with naive CD4+ or CD8+ T cells. It’s important to note that mature CD4+ or CD8+ T cells perform distinct functions in different tumors, underscoring the complexity and diversity of the tumor immune landscape (**Figure 7A**). Except for the macrophages, exhausted T cells were positively correlated with OASL in most tumors, particularly in UVM, THCA, SKCM, KIRC and KICH (R > 0.5). However, a negative correlation with OASL was noted in certain tumors, including ACC, CHOL, MESO, READ and PAAD, as illustrated in **Figure 7B**. Further investigation into the co-localization of OASL and CD68 (a classic biomarker of non-specific macrophage) in the single-cell database demonstrated a significant positive correlation in PAAD (R = 0.64, *p* < 0.001) and LIHC (R = 0.38, *p* < 0.001) (**Figures 7C, D**).

During our analysis of the enrichment of immune-regulatory pathways, we observed positive correlation between OASL expression and classic anti-tumor immune pathways across various tumors (**Figure 7E**). Delving deeper into immune checkpoints related indicators and pathways, we discovered that the correlation between OASL and PD-L1 was predominantly positive, though a noticeable negative relationship was obverse in PAAD, GEM and ACC (**Figure 7F**). It is worthy that STAT1, as a key transcription factor, plays a crucial role in the immune response of IFN signaling. STAT1 is activated when IFN binds to its receptor, which in turn affects the expression of PD-L1. As illustrated in **Figure 7G**, our results also observed a strong positive correlation between the expression of OASL and STAT1 (R = 0.4, *p* < 0.001). Other anti-inflammatory pathways that can activate PD-L1 such as INF-γ response and IL6_JAK_STAT3 signaling, leading to an immunosuppressive state, also show a positive correlation with OASL (**Figures 7H, I**).

In summary, high expression of OASL in tumor tissues activates immune responses such as IFN-γ, STAT1, IL6_JAK_STAT3 signaling, which in turn promotes the expression of PD-L1. This leads to T cell exhaustion, creating an immunosuppressive state and ultimately facilitating tumor immune escape.

## 4 Discussion

This study comprehensively investigated the expression and prognosis of OASL across various cancers. We found that OASL is overexpressed in several cancer types and is associated with poor prognosis. Notably, OASL exhibits diverse and even opposite roles in different cancers. For instance, OASL expression indicates worse prognosis in cancers such as PAAD, KIRC, LGG, THYM, and UVM, but suggests better function in BLCA and SKCM, aligning with previous findings (30–32). This variability may stem from tumor heterogeneity and the complexity of the TME. Additionally, we delved into the types of genetic variations in OASL, especially those mutations affecting transcription factors or regulatory regions, which could contribute to tumor heterogeneity (34, 35). Among SNP variations, nonsense mutations occurred most frequently. CNVs are considered one of the drivers of tumor progression (36). As noted by *Stratton et al.,* amplification of CNVs in specific regions may lead to overexpression of genes within those areas (34). This is consistent with our findings that OASL CNVs can affect the prognosis of COAD, DLBL, and SARC.

In the field of cancer treatment, there have been a revolution with tumor medications transitioning from traditional chemotherapy and radiotherapy to immunotherapies during past few decades (2, 3, 37). ICB therapy, the first generation of antibody-based immunotherapy, catalyzes the immune system’s attack on tumors by inhibiting checkpoint interactions between tumors and immune cells (38). ICB therapy has proven effective in treating a range of cancers, including liver cancer (39), melanoma (40, 41), non-small cell lung cancer (42, 43), renal cell carcinoma (44, 45), and bladder cancer (46, 47). As researches progress, its application continues to expand steadily. ICB therapy inhibits tumor growth by blocking ICs and enhancing anti-tumor T cell activity. It is well-known that the therapeutic efficacy is associated with these characters, including high tumor mutation burden (TMB-H) (48), mismatch repair deficiency (dMMR) (49), or significant microsatellite instability (MSI) (50). Cancer cells with these traits can produce more neoantigens, activating an immune response and creating a “hot” TIME (51). *Zhang et al.* found that the purity of gliomas and the associated non-tumor cells in the TME have significant clinical, genomic, and biological implications (52). As further shown in *Rhee’s* study, the expression of immunotherapy-related genes and specific immune genes, as well as the abundance of immune cell infiltration, are negatively correlated with tumor purity (53). Similarly, we assessed genomic heterogeneity indicators (especially in purity), finding that OASL expression is significantly negatively with purity across tumors. These findings further demonstrate the OASL could participant in the forming of TIME, and plays significant impact on tumor heterogeneity and biological characteristics.

Based on these results, we conclude there is a certain correlation between OASL and the TIME, which has been validated at both the pan-cancer and PAAD levels. Various immune cells infiltrate the TIME, among which CTLs are crucial for immune surveillance. By recognizing and targeting tumor cells through the TCR receptor and through the release of cytotoxic factors and secretion of cytokines, CTLs can not only directly kill tumor cells but also regulate the TME to inhibit tumor growth and spread (54). Studies have shown that high abundance of CTLs with a killing effect in tumors is a prognostic indicator, with both positive (55) and negative (56) implications. Actually, the prognostic value of tumor-infiltrating lymphocytes (TILs) also depends on their specific subsets and the balance between them within TIME (57, 58). However, our findings suggest that high expression level of OASL weakened or even reversed the beneficial effect, consistent with previous studies that reported ISG expression could affect CTL activity (59, 60).

*Benci’s* research identified ISG resistance signature (ISG.RS) as a collection of ISGs associated with resistance to ICB treatment, noting its high expression mainly in cancer cells (61). As part of the anti-viral defense mechanisms in innate immunity, the expression level and activity of OASL varies in different cells based on cell type, tissue environment, and infection status. Hence, we further investigated the expression source of OASL in tumor as an ISG. EPCAM, a cell adhesion molecule, is mainly considered a cancer cell surface marker and therapeutic target (62). Single-cell analysis confirmed a positive correlation between the expression of OASL and EPCAM, and IHC verified high OASL expression in the PAAD cohort. It is suggested that over expressed OASL is related to cancer cells themselves in the TIME, leading to immune response of tumor cells. Consequently, we further observed an increase in representative immune checkpoints like CD274/PD-L1, LGALS9/Galectin-9, and IDO1 in tumors, which was obviously positive correlated with OASL. It’s easily inferred that tumor cell itself induces PD-L1 overexpression by upregulating OASL, affecting T cell dysfunction and leading to exhaustion.

Specific mechanisms have been revealed for the regulatory relationship between PD-L1 upregulation and T cell exhaustion in tumor cells, mainly involving several key signaling pathways (63). Our research demonstrates that OASL upregulation is closely related to IFN-γ, STAT1, and IL-6/JAK/STAT3 pathway. In agreement with ours, studies have shown that STAT1 promotes PD-1/PD-L1 expression in the TIME, thereby affecting immune suppression (64–66). IL-6 promotes PD-L1 expression in human hepatocellular carcinoma (HCC) through the JAK/STAT3 signaling pathway, thereby reducing the expression of protein tyrosine phosphatase receptor and further promoting tumor immune escape (67). *Yu* and *Johnson et al.* illustrated the IL-6/JAK/STAT3 pathway has become an important target in cancer and inflammatory disease research due to its crucial role in tumor progression and immune escape (68, 69). Additionally, IFN-γ participates in regulating the expression of PD-L1 through multiple signaling pathways (70). This process not only involves direct transcription factors such as NF-κB and HIF-1α, but also includes other factors (oxidative stress-related and ISGs), which all indirectly affects the expression of PD-L1 (71). These results suggest that the expression of immune checkpoints like PD-1/PD-L1 were caused by immune response pathways, leading to tumor immune escape.

The important role of TAMs within TIME has been reported a lot (72). Their function can be broadly categorized into pro- or anti-tumorigenic activities, which largely dependent on their polarization state. In general, M1 macrophages exhibit pro-inflammatory and anti-tumor effects, but TAM in the TIME often polarize to a phenotype (M2) that supports tumor growth (73, 74). M2 release cytokines and growth factors such as IL-10 and TGF-β, which inhibit the functions of CTL and support immune evasion. We attempted to explore the connection between the expression of OASL and macrophages. It is found that the expression of OASL was positively correlated with the abundant infiltration of macrophages in overall types of tumors. Through single-cell data, we also verified the co-expression of OASL and the macrophage marker CD68 in PAAD and LIHC. These data indicate that tumor cells may affect macrophage’s content and function through elevated expression of OASL. Unfortunately, CD68 is generally regarded as a marker for non-specific macrophage but not specifically for M2 phenotype. Therefore, how the improved OASL affects M2 for promoting tumorigenesis still requires further exploration.

To summarize, our study demonstrates the possibility that OASL could serve as a TIME-related immunotherapeutic target. The observed positive correlation between OASL and PD-L1 suggests that targeting OASL could enhance the efficacy of PD-1/PD-L1 inhibitors, synergizing tumor immune-targeted therapy. However, there are still some limitations about this research. The interaction between OASL and immune cells within TIME is not fully elucidated, and the heterogeneous roles of OASL across tumors require further *in vitro* and *in vivo* investigation. We look forward to in-depth research on tumor immunology concerning with TIME. The advancements in this area may offer new hope for patients resistant to cancer treatment, which also contributing to the development of personalized cancer therapy approaches.

## 5 Conclusion

Our study comprehensively explored OASL’s role in cancer and its interaction with the TIME opens new avenues for potentially leading to more effective and personalized cancer treatments.

## Data Availability

The original contributions presented in the study are included in the article/**Supplementary Material**. Further inquiries can be directed to the corresponding authors.
