# Supplementary material for: Multi-omics landscape of Interferon-stimulated gene OASL reveals a potential biomarker in pan-cancer: from prognosis to tumor microenvironment
Source: Front Immunol. 2024 Sep 2;15:1402951. doi: 10.3389/fimmu.2024.1402951 (PMC11402691; doi:10.3389/fimmu.2024.1402951)
Supplement: Supplementary file 1 [file Table1.docx]

**[Multi-omics landscape of interferon-stimulated gene OASL reveals a potential biomarker](https://pubmed.ncbi.nlm.nih.gov/36348462/)**

**in pan-cancer: from prognosis to tumor microenvironment**

*Yi Liu ^†,1^, Runyu Yang ^†,1^, Mengyao Zhang ^†,1^, Bingyu Yang ^1^, Yue Du ^1^, Hui Feng ^1^, Wenjuan Wang ^1^, Busheng Xue ^*,1^, Fan Niu ^*,1^, Pengcheng He ^*,1^*

^1^ Department of Hematology, The First Affiliated Hospital of Xi’an Jiaotong University, No. 277 Yanta West Road, Xi'an, 710061 Shaanxi, China

E-mail: [hepengcheng@xjtu.edu.cn](mailto:hepengcheng@xjtu.edu.cn); [niufan@xjtufh.edu.cn](mailto:niufan@xjtufh.edu.cn); bushengxue@xjtufh.edu.cn

† These authors contributed equally to this work.

* Corresponding authors.

**Keywords:** OASL; pan-cancer analysis; tumor immune microenvironment; T cell dysfunction; biomarker

**Table S1.** The siRNA sequences.

| **siRNA Name** | **Sequence (5'to3')** |
| --- | --- |
| human-OASL-siRNA-1-F | GAGAAAUUUCGUGAAACAUTT |
| human-OASL-siRNA-1-R | AUGUUUCACGAAAUUUCUCTT |
| human-OASL-siRNA-2-F | CCUUAUGAGCCCAUAAGGATT |
| human-OASL-siRNA-2-R | UCCUUAUGGGCUCAUAAGGTT |
| human-OASL-siRNA-3-F | CAAUCAUUGAGGAUUGUGUTT |
| human-OASL-siRNA-3-R | ACACAAUCCUCAAUGAUUGTT |

**Table S2.** The primer sequences for qPCR assays.

| **Primer name** | **Primer sequence (5'to3')** |
| --- | --- |
| h-GAPDH-F | CTGGGCTACACTGAGCACC |
| h-GAPDH-R | AAGTGGTCGTTGAGGGCAATG |
| h-OASL-F | AAAAGAGAGGCCCATCATCCTG |
| h-OASL-R | ACTGTCAAGTGGATGTCTCGTG |

**Table S3.** The Abbreviations of all cancers in TCGA database.

| **Abbreviations** | **Full name** |
| --- | --- |
| ACC | Adrenocortical carcinoma |
| BLCA | Bladder urothelial carcinoma |
| BRCA | Breast invasive carcinoma |
| CESC | Cervical squamous cell carcinoma and endocervical adenocarcinoma |
| CHOL | Cholangiocarcinoma |
| COAD | Colon adenocarcinoma |
| DLBC | Lymphoid neoplasm/difuse large B-cell lymphoma |
| ESCA | Esophageal carcinoma |
| GBM | Glioblastoma multiforme |
| HNSC | Head and neck squamous cell carcinoma |
| KICH | Kidney chromophobe |
| KIRC | Kidney renal clear cell carcinoma |
| KIRP | Kidney renal papillary cell carcinoma |
| LAML | Acute myeloid leukemia |
| LGG | Brain lower grade glioma |
| LIHC | Liver hepatocellular carcinoma |
| LUAD | Lung adenocarcinoma |
| LUSC | Lung squamous cell carcinoma |
| MESO | Mesothelioma |
| OV | Ovarian serous cystadenocarcinoma |
| PAAD | Pancreatic adenocarcinoma |
| PCPG | Pheochromocytoma and paraganglioma |
| PRAD | Prostate adenocarcinoma |
| READ | Rectum adenocarcinoma |
| SARC | Sarcoma |
| SKCM | Skin cutaneous melanoma |
| STAD | Stomach adenocarcinoma |
| TGCT | Testicular germ cell tumors |
| THCA | Thyroid carcinoma |
| THYM | Thymoma |
| UCEC | Uterine corpus endometrial carcinoma |
| UCS | Uterine carcinosarcoma |
| UVM | Uveal melanoma |

**Table S4.** The information of Single cell-RNA seq datasets involved in this study.

| **Dataset** | **Sample number** | **Cell number** | **Description** | **Ref.** |
| --- | --- | --- | --- | --- |
| CRA001160 | 35 | 57443 | Single cell-RNA seq data PAAD Patients | 31273297 |
| GSE161529 | 52 | 332168 | Single cell-RNA seq data BRCA Patients | 33950524 |
| GSE172577 | 6 | 57503 | Single cell-RNA seq data HNSC Patients | 34044317 |
| GSE140228 | 5 | 61690 | Single cell-RNA seq data HNSC Patients | 31675496 |
